# Heart Failure is Highly Prevalent and Difficult to Diagnose in Severe Exacerbations of COPD Presenting to the Emergency Department

**DOI:** 10.3390/jcm9082644

**Published:** 2020-08-14

**Authors:** Mariaenrica Tinè, Erica Bazzan, Umberto Semenzato, Davide Biondini, Elisabetta Cocconcelli, Elisabetta Balestro, Alvise Casara, Simonetta Baraldo, Graziella Turato, Manuel G. Cosio, Marina Saetta

**Affiliations:** 1 Department of Cardio-Thoracic-Vascular Sciences and Public Health, University of Padova, 35127 Padova, Italy; mariaenrica.tine@gmail.com (M.T.); erica.bazzan@unipd.it (E.B.); umberto.semenzato@gmail.com (U.S.); dav.biondini@gmail.com (D.B.); ecocconcelli@icloud.com (E.C.); elisabetta_balestro@hotmail.com (E.B.); alvise.casara@gmail.com (A.C.); simonetta.baraldo@unipd.it (S.B.); graziella.turato@unipd.it (G.T.); manuel.cosio@mcgill.ca (M.G.C.); 2 Meakins-Christie Laboratories, Respiratory Division, McGill University, Montreal, QC H4A3J1, Canada

**Keywords:** severe exacerbations, hearth failure, COPD mortality, blood lymphocytes, COPD care program

## Abstract

Background: Some 20% of patients with stable Chronic Obstructive Pulmonary Disease (COPD) might have heart failure (HF). HF contribution to acute exacerbations of COPD (AECOPD) presenting to the emergency department (ED) is not well established. Aims: To assess (1) the HF incidence in patients presenting to the ED with AECOPD; (2) the concordance between ED and respiratory ward (RW) diagnosis; (3) the factors associated with risk of death after hospital discharge. Methods: Retrospective chart review of 119 COPD patients presenting to ED for acute exacerbation of respiratory symptoms and then admitted to RW where a final diagnosis of AECOPD, AECOPD and HF and AECOPD and OD (other diagnosis), was obtained. ED and RW diagnosis were then compared. Factors affecting survival at follow-up were investigated. Results: At RW, 40.3% of cases were diagnosed of AECOPD, 40.3% of AECOPD and HF and 19.4% of AECOPD and OD, with ED diagnosis coinciding with RW’s in 67%, 23%, and 57% of cases respectively. At RW, 60% of patients in GOLD1 had HF, of which 43% were diagnosed at ED, while 40% in GOLD4 had HF that was never diagnosed at ED. Lack of inclusion in a COPD care program, HF, and early readmission for AECOPD were associated with mortality. Conclusions: HF is highly prevalent and difficult to diagnose in patients in all GOLD stages presenting to the ED with severe AECOPD, and along with lack of inclusion in a COPD care program, confers a high risk for mortality.

## 1. Introduction

Chronic obstructive pulmonary disease (COPD), whose main risk factors are alpha1 antitrypsin deficiency and cigarette smoking, is characterized by an inflammatory reaction to the inciting agent, such as cigarette smoking, that, by destroying lung airways and parenchyma, progressively diminishes the ventilatory capacity [1,2].

About 75% of patients with COPD over 10 years [3] might suffer from acute exacerbations of their disease (AECOPD), characterized by symptomatic deterioration with increased dyspnoea, cough and sputum beyond the day to day variation. These episodes might lead to a change in the medical treatment in moderate AECOPD or to emergency department (ED) visits and hospitalization in severe AECOPD [1]. Reported rates of AECOPD and severe AECOPD vary dramatically based on the definition used, different methods of data collection and underreporting of AECOPDs. Rates ranging from 0.8 to 3.8/year in moderate AECOPD and from 0.15 to 0.25/year in severe AECOPD have been reported [4]. AECOPD dramatically impact on patients’ quality of life [5] and it has been shown that, among patients with severe exacerbations, one out of eight discharged from a first COPD hospitalization might die within the following year [6].

COPD is entangled in a network of other chronic conditions which may additionally impact the patient and the healthcare system [7]. As such, COPD is considered as a component of a multimorbid condition often affecting older sedentary smokers [8]. Patients with COPD often have associated comorbid conditions, principally cardiovascular, metabolic, and musculoskeletal [7,8,9]. Several of these comorbidities may present with acute events mimicking or contributing to the symptoms of presentation and to the morbidity and mortality of AECOPD. About 20% of patients with COPD have a diagnosed cardiovascular morbidity [10] and it has been estimated that about 20% of AECOPD could be due to acute decompensated heart failure [11,12].

Since exacerbations of COPD are defined in the literature only on the basis of symptomatic deterioration, without considering the possible cause, a cardiovascular instability precipitating acute deterioration of symptoms in patients with COPD is probably common. However, diagnosis of cardiac disease in COPD, especially when presenting as an AECOPD, is difficult, particularly in an ED environment, and necessitates a high degree of clinical suspicion [10].

Based on these premises, we performed a retrospective chart review of patients presenting to the ED with AECOPD aimed at: (1) assessing the true incidence of heart failure (HF), as diagnosed in the respiratory ward (RW), in patients with COPD presenting with acute deterioration of symptoms to the ED and then hospitalized; (2) comparing the RW diagnosis with the ED diagnosis; (3) follow-up review to investigate patients outcome, rate of mortality, and the factors associated with risk of death after hospital discharge.

## 2. Methods

### 2.1. Study Design

We performed a retrospective study based in a chart review of 1470 patients presenting to the emergency department (ED) of the University Hospital of Padova, Italy from January 2014 to December 2018 with acute exacerbation of respiratory symptoms deserving admission to the respiratory ward (RW). From this population, we selected 119 smoking subjects (mean age of 74 ± 10 years; of which 66 were males and 53 females) with documented lung function diagnosis of COPD (FEV_1_/FVC post bronchodilator < 0.7) presenting to the ED with acute exacerbation of respiratory symptoms. Patients with a history of asthma were excluded (Figure S1). The clinical characteristics of the population are described in Table 1.

At the ED, blood count, C-reactive protein (CRP), *N*-terminal pro B-type natriuretic peptide (NT-proBNP), and blood gas analysis were done. The diagnosis assigned by the ED physicians for these patients who presented with acute severe exacerbation of respiratory symptoms was obtained from the patients chart and read as: (1) respiratory failure secondary to AECOPD, which included patients diagnosed in the ED of acute exacerbation due to COPD with no other adjunctive causes; (2) respiratory failure secondary to AECOPD and HF, which included patients diagnosed in the ED of acute exacerbation due to COPD and concomitant heart failure; (3) respiratory failure secondary to other diagnosis (OD) which included patients with acute exacerbation of respiratory symptoms thought to be due to other diagnosis than AECOPD or AECOPD and HF (like pneumonia, pulmonary emboli, or “respiratory failure” with non-specified cause by ED physicians). We used an abbreviated form of the original ED diagnosis and called the groups AECOPD, AECOPD and HF and AECOPD and OD (other diagnosis). Brief definition of the diagnostic groups and laboratory tests available at ED and RW, are shown in Supplementary Materials and Table S1.

### 2.2. Respiratory Ward Admission

Once admitted to the RW, the diagnostic assessment included: clinical history and examination, microbiological assays and blood count, C-reactive protein (CRP), *N*-terminal pro B-type natriuretic peptide (NT-proBNP), lung function, chest X-rays and CT scans for the presence of bronchiectasis and emphysema, assessment of co-morbidities, and echocardiography that was deemed indicated in 76 cases and was positive for HF in 48. The use of respiratory and cardiovascular medications and the use of long-term home oxygen therapy (LTOT) and home non-invasive ventilation (NIV) were recorded. Patients were evaluated and managed by a pneumology team who eventually formulated the final discharge diagnosis. These RW diagnosis were then compared to those formulated at ED. After discharge all patients were invited to be followed in the COPD care program in our institution with scheduled visits at 1, 3, and 6 months after discharge.

### 2.3. Follow-Up after Discharge from Chart Review

After discharge patients were followed until November 2019 (mean follow-up 2.2 ± 1.7 years) to assess recurrent COPD exacerbations and/or survival. Clinical, functional, and laboratory data assessed during hospitalization were investigated to identify potential factors affecting survival in our population.

The study conformed to the Declaration of Helsinki. The protocol was approved by the Ethics Committee of the Padova City Hospital.

### 2.4. Statistics

Comparisons among groups were evaluated with Kruskal–Wallis and Mann–Whitney U-tests. Distributions of categorical variables were compared with the Chi-squared test or Fisher’s exact test when the sample size was small (*n* < 5). Analyses of overall survival were performed using Kaplan–Meier survival curves. Cox proportional risk regression model was used to evaluate independent prognostic factors (Supplementary Data). To assess the ED diagnosis accuracy compared to the RW diagnosis, sensitivity, specificity, positive (PPV) and negative predictive values (NVP) and positive and negative likelihood ratios (LR+ and LR−) were calculated (Details on Supplementary Materials).

## 3. Results

Of the 119 patients who met the eligibility criteria for the study, at the ED 53% were diagnosed as AECOPD, 11% as AECOPD and HF and 36% as AECOPD and OD. At discharge from the RW, 40.3% were diagnosed as AECOPD, 40.3% as AECOPD and HF and 19.4% as AECOPD and OD (Details on Supplementary Data). Table 1 shows that the clinical characteristics of the whole population grouped by RW discharge diagnosis were similar in all groups except for long-term oxygen therapy (LTOT), that was used significantly more by patients with AECOPD and HF than with AECOPD and OD (*p* = 0.005), and the number of comorbidities, that was higher in AECOPD and HF than in AECOPD (*p* = 0.01). The use of respiratory and cardiac medications was similar in all groups except anticoagulants, more often used in patients with AECOPD and HF (Supplementary Materials, Tables S2 and S3).

### 3.1. Diagnostic Comparison between ED and RW

**Figure 1:**
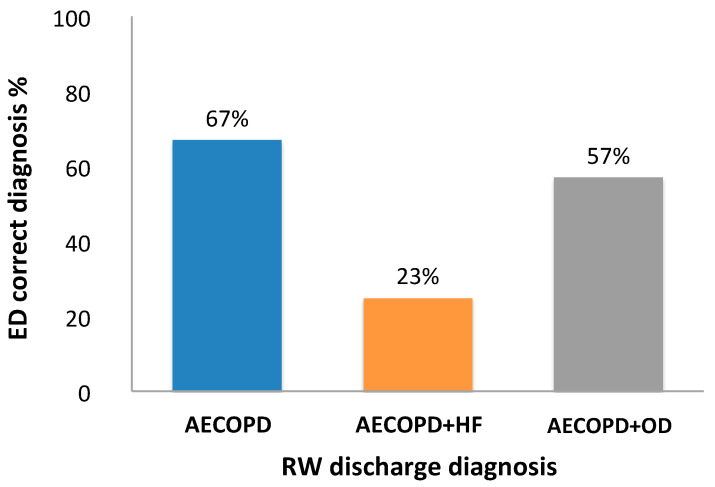
Comparison of Emergency Department (ED) and Respiratory Ward (RW) diagnosis. Percentage of ED diagnosis that coincided with the RW diagnosis of acute exacerbation of COPD (AECOPD), acute exacerbation of COPD and heart failure (AECOPD and HF) and diagnosis other than AECOPD or AECOPD and HF (AECOPD and OD).

When ED and RW diagnosis were compared (Figure 1), ED diagnosis of AECOPD coincided with the RW diagnosis (true diagnosis) in 67% of cases, ED diagnosis of AECOPD and HF coincided with RW diagnosis in 23% of cases, and ED diagnosis of AECOPD and OD coincided with RW diagnosis in 57% of cases.

Both the sensitivity (27%, 23%, 52% for AECOPD, AECOPD and HF, and AECOPD and OD respectively) and the specificity of the ED diagnosis were poor (56% for AECOPD and 69% for AECOPD and OD) except for the specificity of the AECOPD and HF that was high (99%, 11 out of 12 diagnosis were correct). The limited diagnostic accuracy of the ED diagnosis was confirmed by other performance values (PPV, NPV, LR+ and LR−) as detailed in Table 2.

**Figure 2:**
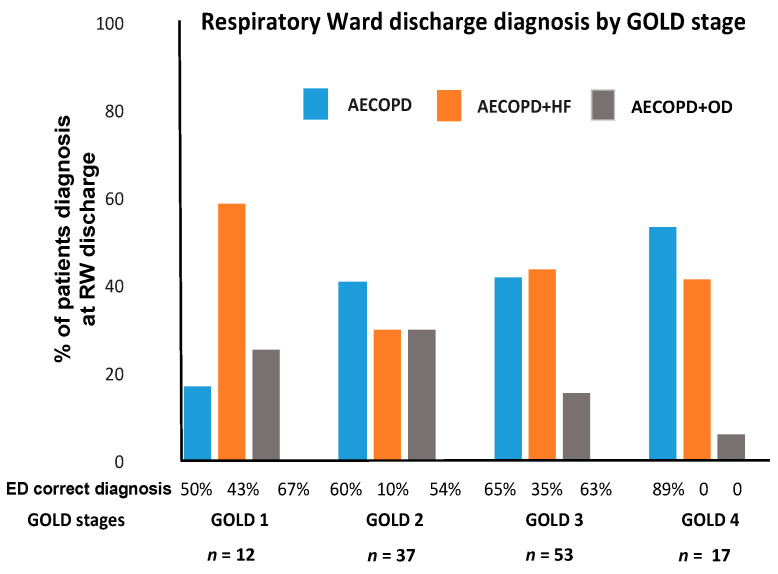
Prevalence of AECOPD type by RW diagnosis in GOLD stages and coincidence with ED diagnosis. Percent of patients diagnosed at the RW of AECOPD, AECOPD and HF, AECOPD and OD in the different GOLD stages. Bottom of the figure shows the percentage of correct diagnosis at the ED for each RW diagnosis.

Figure 2 shows the concordance in diagnosis between ED and RW tabulated and expressed by increasing severity of COPD, from GOLD1, mild, to GOLD4, very severe disease. In GOLD1, AECOPD and HF was the prevalent diagnosis and it was correctly diagnosed at ED in 43% of cases. In GOLD4, both AECOPD and AECOPD and HF were prevalent diagnoses but, while AECOPD was correctly diagnosed at ED in 89% of cases, the presence of AECOPD and HF was never diagnosed in ED. In GOLD2 and 3, AECOPD and AECOPD and HF diagnoses were similarly represented but even in these stages, the HF component was difficult to recognize at ED.

### 3.2. Laboratory Results

White blood cell count, neutrophil count, and blood gases at ED admission were similar in the 3 RW diagnostic groups (Supplementary Materials, Table S4). They were similar also when the 3 ED diagnostic groups were considered.

Table 3 shows the values of the NT-proBNP and CRP done at the ED (44 of 119 patients) and during RW admission (119 of 119 patients) according to the RW diagnostic groups. NT-proBNP and CRP were significantly higher in AECOPD and HF than in AECOPD (*p* < 0.0001) and similar in AECOPD and HF and AECOPD and OD. CRP was higher both in AECOPD and OD and AECOPD and HF than in AECOPD (*p* = 0.006; *p* = 0.04 respectively). The test results from the RW and from ED were similar.

The laboratory data at discharge from the RW showed no differences in the white blood cells count except for the lymphocytes number, which was significantly lower in AECOPD and HF than in AECOPD and OD (*p* = 0.01) and tended to be lower than in AECOPD (*p* = 0.07). The neutrophil-to-lymphocyte ratio (NLR) was higher in AECOPD and HF (*p* = 0.05), most likely due to the low blood lymphocyte count in this group (Supplementary Data, Table S5).

### 3.3. Hospitalization

The mean length of hospital stay (9 ± 6 days) did not differ in the 3 groups. During hospitalization a higher number of patients with AECOPD and HF (28/48, 58%) required non-invasive ventilation than those with AECOPD (15/48, 31%; *p* = 0.0001) and AECOPD and OD (7/23, 30%; *p* < 0.0001) (Table S5). Among the 55 patients who underwent microbiological assays, 38 (69%) were positive for either bacterial or viral pathogens with no difference among groups (Supplementary Data, Table S5).

### 3.4. Post Discharge Follow-Up

**Figure 3:**
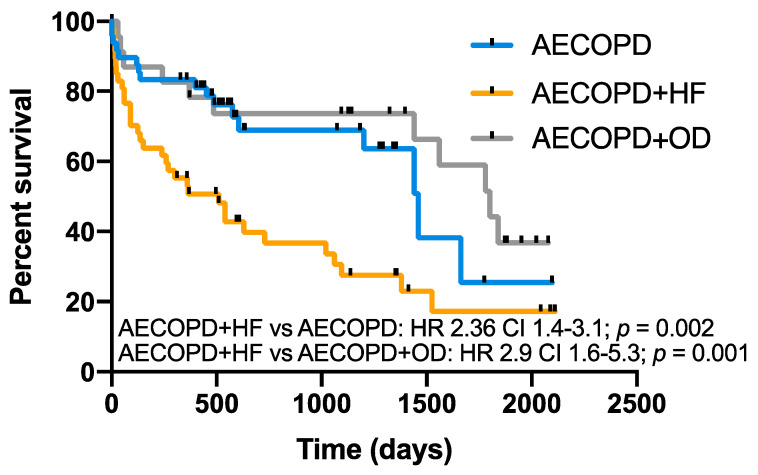
Long-term survival according to RW diagnosis. Kaplan–Meier plots showing survival in COPD patients with AECOPD, AECOPD and HF and AECOPD and OD over the follow-up period (overall comparison *p* = 0.001). AECOPD and HF had lower survival compared to AECOPD (*p* = 0.002) and AECOPD + OD (*p* = 0.001).

**Figure 4:**
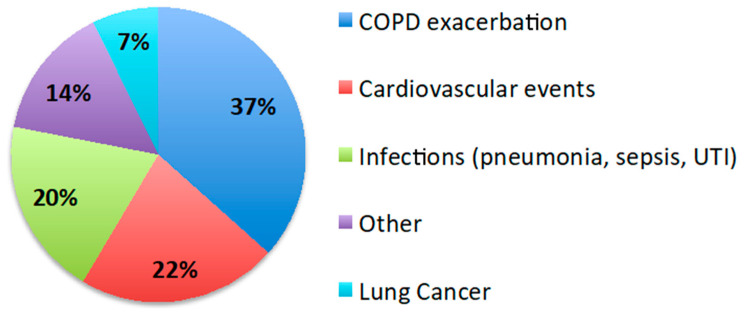
Causes of death. Pie chart showing the causes of death during the follow-up period. The most frequent causes were COPD exacerbations and cardiovascular events.

The mean follow-up period after discharge was 2.2 ± 1.7 years. Forty percent (48/119) of patients were enrolled in the COPD care program of our institution. Thirty percent (36/119) of patients died within the first year after discharge and 51% (61/119) by the end of follow-up. The survival of the AECOPD and HF group was worse than that of AECOPD (HR 2.36, 95% CI 1.4–3.1; *p* = 0.002) and AECOPD and OD groups (HR 2.9, 95% CI 1.6–5.3; *p* = 0.001), while there was no difference between AECOPD and AECOPD and OD (Figure 3). The most prevalent causes of death were COPD exacerbation (37%) and cardiovascular events (22%) (Figure 4).

**Figure 5:**
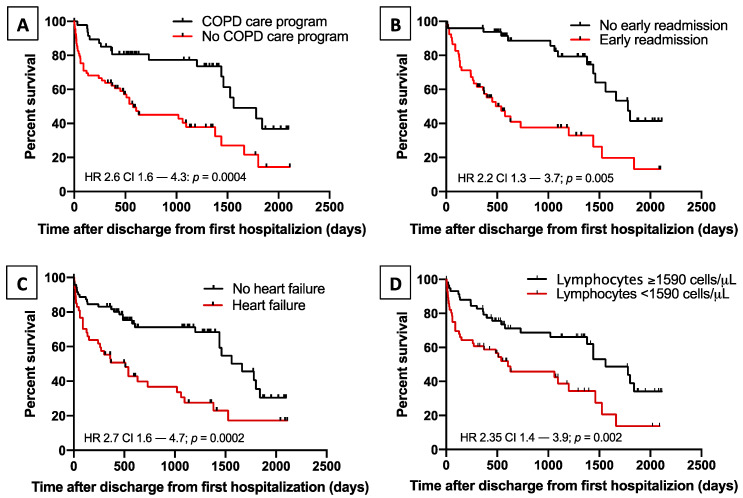
Risk factors for mortality. Kaplan–Meier plots showing the effect on survival of: lack of inclusion in a COPD care program (*p* = 0.0004, panel (**A**)); early readmission for COPD exacerbation (*p* = 0.005, panel (**B**)); heart failure at hospitalization (*p* = 0.0002, panel (**C**)); and low blood lymphocytes count (*p* = 0.002, panel (**D**)).

Cox regression analysis showed that among all the variables considered, lack of inclusion in a COPD care program (HR 2.56 *p* = 0.006), heart failure (HR 2.19 *p* = 0.005), early readmission for COPD exacerbation (HR 2.05 *p* = 0.02), and low lymphocyte count (HR 0.57 *p* = 0.01) were independent risk factors for death after discharge from RW (Figure 5, Table 4, and Supplementary Materials, Table S6).

## 4. Discussion

As part of the multimorbid condition associated with age and smoking, COPD is often associated with cardiac and other comorbidities [7,8,9]. Cardiovascular disease can be found in about 20% of patients with COPD, and due to the similarity of their clinical presentation, could be extremely difficult to diagnose [1,10,11,13,14].

The frequency of cardiac decompensation and its importance as a factor contributing to the clinical presentation of severe AECOPD, has been recognized but has never been directly investigated [1]. Our study shows the high prevalence of cardiac decompensation in COPD patients presenting with severe AECOPD, and especially underlines the difficulty of its diagnosis in the ED. In our population, cardiac failure, along with lack of inclusion in a COPD care program, conferred a high risk of mortality. Importantly, a cardiac cause for AECOPD can be present with all degrees of COPD severity, from mild to very severe.

We intentionally studied only patients with known COPD diagnosed by pulmonary function tests, in order to avoid the incorporation of patients over-diagnosed and under-diagnosed of COPD, often present in large clinical databases, which could change the reality of the situation [15,16,17,18].

A component of heart failure, AECOPD and HF, at the ED was diagnosed or suspected in only 23% of the HF cases proven after admission, a surprising discrepancy which highlights the difficulties in diagnosing HF in the presence of known COPD. It is possible that, once the decision for admission by the ED physician had been taken, most likely based on the severity of the patient condition, finessing the precise diagnosis for the cause of symptom deterioration became a secondary issue.

It is well known that the presence and number of comorbidities in smokers with COPD are independent from the degree of severity of the disease [19,20], a fact well illustrated, at least for cardiac comorbidities, in our population. HF was diagnosed in 60% of patients presenting with very mild disease, GOLD1, of which only 43% were correctly diagnosed at the ED (Figure 2). In patients with very severe disease, GOLD4, HF was present in 40% of our cases but never diagnosed in the ED (Figure 2). These findings go along with previous reports stating that cardiovascular disease can be detected in as many as 55% of patients admitted with AECOPD [11,21,22,23].

The strong association of underlying cardiovascular comorbidities in any degree of COPD severity suggests that ED management should always include a workup for potential associated cardiovascular events. A high degree of suspicion, past history of cardiovascular diseases, ECG and NT-proBNP, a valuable tool in the diagnosis of HF, are recommended steps to reach a diagnosis of HF, yet it will still be difficult in the presence of COPD [13,24]. In our cohort, the results of NT-proBNP and CRP done during the RW admission, where heart failure was confirmed by the echocardiogram, were similar to the ones done in the ED. A high value of NT-proBNP could be of help in identifying a heart failure component in COPD patients presenting with severe exacerbations of respiratory symptoms.

The RW admission diagnostic procedures including cardiac echo revealed the high frequency of cardiac failure as a cause for the acute deterioration of respiratory symptoms in these patients. None of the cardiac complication that have been reported to occur in AECOPD after admission, such as myocardial infarction or arrhythmias [11,24], occurred in our population, which confirms that cardiac failure, when present, was part of the presenting AECOPD. Several mechanisms such as tachycardia, hypoxemia, increased pulmonary artery pressure, infective exacerbations provoking arterial stiffness have been postulated as causing cardiac distress during AECOPD in subjects with associated cardiac disease [25,26]. In addition, lung abnormalities secondary to COPD such as emphysema and hyperinflation, by raising intrathoracic pressures, can decrease both preload and afterload, reduce biventricular filling and provoke diastolic dysfunction, contributing significantly to cardiac dysfunction in these patients [27]. Interestingly, dual bronchodilation with indacaterol–glycopyrronium, by diminishing hyperinflation, significantly improves cardiac function, as measured by left-ventricular end-diastolic volume, in patients with COPD [27]. In contrast to the described beneficial effects of bronchodilation with indacaterol–glycopyrronium, in comorbid cardiorespiratory disease high doses of β2 agonists have been associated with an increase in mortality in COPD exacerbations [28,29,30], and ought to be used with care in AECOPD with an associated cardiac component.

In the numerous important population studies analyzing both moderate and severe exacerbations in patients with COPD, the possible different causes or possible triggering factors for the exacerbation are not investigated [3,31,32,33]. The results of these studies are useful as far as understanding the magnitude of the problem but not the problem itself and perpetuate the idea of AECOPD as a single entity. This situation is even of greater concern when the term AECOPD, which in a substantial proportion of cases is not a real AECOPD, is used to investigate treatment modalities.

Since all the patients in the cohort survived the AECOPD episode, we were able to review their outcome and document the risk factors and causes of mortality. Thirty percent of the subjects died within the first year after discharge, while 51% were dead by the end of the follow-up. In our analysis the most important risk factors for mortality were the lack of inclusion in a COPD care program along with heart failure, early readmission, and low blood lymphocyte counts, as it has been shown before [34].

The relatively low number of patients is a limitation to the study, particularly for the comparison of three groups. However, the low prevalence of severe AECOPD and the strict conditions for patient selection, which required a documented diagnosis of COPD and admission to the respiratory ward, resulted in 119 patients selected in four years. Similarly, being a single center study is another limitation of our study, yet the aims are original, and the interesting results might stimulate further research in this area with larger prospective studies. However, the retrospective analysis in our specific study was in our view appropriate to answer our aims, since a prospective study with the ED would have not provided the sought-after answer.

## 5. Conclusions

Our findings highlight that a so-called severe AECOPD is often due or associated with unsuspected and difficult to detect cardiac decompensation in about 40% of cases and with other disparate causes in 20%. In agreement with Fabbri and co-workers [15] we believe that the term “exacerbation of COPD” should be changed to “Exacerbations of symptoms in a patient with COPD” in order to stimulate a careful investigation of the complex respiratory and non-respiratory mechanisms potentially involved and treat accordingly. Furthermore, cardiac failure, along with lack of inclusion in a COPD care program, confers a high risk of mortality.

## Figures and Tables

**Table 1:** Clinical characteristics in the whole population and in the three RW diagnostic groups.

		Respiratory Ward Diagnostic Groups			
	Whole Population	AECOPD (*n* = 48)	AECOPD + HF (*n* = 48)	AECOPD + OD (*n* = 23)	*p*
Subjects, *n* (%)	119 (100)	48 (40.3)	48 (40.3)	23 (19.4)	-
Age, years	74 ± 10	72 ± 10	76 ± 9	76 ± 11	n.s.
Male sex, *n* (%)	66 (55)	30 (63)	22 (46)	14 (61)	n.s.
Smoking History, pack-years	40 ± 24	42 ± 30	40 ± 19	39 ± 20	n.s.
FEV_1_, L	1.08 ± 0.4	1.09 ± 0.5	1.08 ± 0.4	1.12 ± 0.33	n.s.
FEV_1_, % predicted	51 ± 18	48 ± 19	54 ± 21	54 ± 12	n.s.
Presence of					
emphysema, *n* (%)	54 (45)	25 (52)	19 (40)	10 (43)	n.s.
GOLD stage, *n* (%)					
1	12 (10)	2 (4)	7 (14)	3 (13)	n.s.
2	37 (31)	15 (31)	11 (23)	11 (48)	n.s.
3	53 (45)	22 (46)	23 (48)	8 (35)	n.s.
4	17 (14)	9 (19)	7 (15)	1 (4)	n.s
Comorbidities, *n*	3.5 ± 1.8	3 ± 1.7 *	4 ± 1.9	3.5 ± 1.8	0.04
NIV at home, *n* (%)	16 (15)	6 (13)	9 (19)	1 (4)	n.s.
LTOT at home, *n* (%)	66 (55)	24 (50)	34 (70)	8 (35) *	0.01

Data are expressed as number (%) or mean ± SD. *p* values refer to Kruskal–Wallis test or χ^2^ test or Fisher exact test. * significantly different than AECOPD and HF (*p* < 0.01 for each comparison). n.s.: non-significant. AECOPD = acute exacerbation of COPD; AECOPD and HF = acute exacerbation of COPD and heart failure; AECOPD and OD = diagnosis other than AECOPD or AECOPD and HF; ICS = inhaled corticosteroids; OCS = oral corticosteroids; GOLD = Global initiative for Obstructive Lung Disease; NIV = non-invasive ventilation (both non-invasive positive pressure ventilation and continuous positive airway pressure were included); LTOT = long-term oxygen therapy, ACE = angiotensin converting enzyme.

**Table 2:** Diagnostic accuracy of ED diagnosis in relation to RW diagnosis.

ED Diagnosis	Sensitivity (%)	Specificity (%)	PPV (%)	NPV (%)	LR+	LR−
AECOPD	27.0	56.0	51.0	7.0	0.5	1.6
AECOPD + HF	23.0	99.0	92.0	65.0	23.0	0.8
AECOPD + OD	52.0	69.0	28.0	85.0	1.7	0.9

PPV = positive predictive values; NVP = negative predictive values; LR + = positive likelihood ratio; LR− = negative likelihood ratio. AECOPD = acute exacerbation of COPD; AECOPD and HF = acute exacerbation of COPD and heart failure; AECOPD and OD = diagnosis other than AECOPD or AECOPD and HF.

**Table 3:** NT-proBNP and CRP values at ED and RW in the whole population and in the three RW diagnostic groups.

		Whole Population	AECOPD (*n* = 48)	AECOPD + HF (*n* = 48)	AECOPD + OD (*n* = 23)	*p*
Drawn at ED	NT-proBNP, ng/L	1596 ± 2558	144 ± 113 *	2567 ± 2826	1747 ± 2987	0.01
	CRP, mg/L	82.3 ± 99.4	28.6 ± 58.5 *^,§^	93 ± 98.3	132 ± 116.3	0.01
Drawn at RW	NT-proBNP, ng/L	960 ± 2168	140 ± 207 *^,§^	1629 ± 2941	779 ± 895	<0.0001
	CRP, mg/L	60.3 ± 76.2	38.2 ± 58.7 *^,§^	68.3 ± 78.5	89.7 ± 92.3	0.01

Data are expressed as mean ± SD. *p* values refer to Kruskal–Wallis test. * Significantly different than AECOPD and HF (*p* < 0.03 for each comparison). ^§^ Significantly different than AECOPD + OD (*p* < 0.003 for each comparison). At ED, NT-proBNP and CRP were drawn in 44/119 patients. At RW, NT-proBNP and CRP were drawn 119/119 patients. AECOPD = acute exacerbation of COPD, AECOPD and HF = acute exacerbation of COPD and heart failure, AECOPD and OD = diagnosis other than AECOPD or AECOPD and HF; CRP = C-reactive protein; NT-proBNP = *N*-terminal pro B-type natriuretic peptide.

**Table 4:** Risk factors for death, Cox proportional hazards model.

Risk Factor	Univariate Analysis	Cox Regression		
	*p* Value	HR	95% CI	*p* Value
Lack of inclusion in a COPD care program	0.001	2.56	1.31–4.99	0.006
Heart failure	0.0003	2.19	1.26–3.79	0.005
Early readmission for AECOPD	0.006	2.05	1.11–3.79	0.02
Lymphocytes * (cells/µL)	0.0003	0.57	0.37–0.87	0.01
Neutrophil-to-lymphocytes ratio	0.02	-	-	-
GOLD stage 3–4	0.036	-	-	-

Values are expressed as HR (95% CI). Univariate and multivariate Cox proportional hazard regression tests were used to determine the relationship of clinical, functional and serological characteristics with survival. * Lab tests refer to respiratory ward discharge mean value. HR = hazard ratio, CI = confidence interval, AECOPD = acute exacerbation of COPD.

## Supplementary Materials

The following are available online at https://www.mdpi.com/2077-0383/9/8/2644/s1, Figure S1: Patient flow chart, Table S1: Diagnostic tests available at Emergency Department (ED) and Respiratory Ward (RW) and frequency of use in the whole population of 119 patients, Table S2: Medications at baseline in whole population and in the three RW groups, Table S3: Comorbidities in whole population and the three RW groups, Table S4: Laboratory tests at ED visit according to the three RW diagnostic groups, Table S5: Laboratory and clinical data at the RW, Table S6: Risk factors for death after discharge from our RW, non-adjusted and adjusted HR.

## Abbreviations

AECOPD	acute exacerbations of COPD
AECOPD + HF	acute exacerbations of COPD and heart failure
AECOPD + OD	diagnosis other than AECOPD or AECOPD + HF
CRP	C-reactive protein
ED	emergency department
GOLD	Global initiative for Obstructive Lung Disease
HF	Heart failure
ICS	inhaled corticosteroids
LTOT	long-term oxygen therapy
NIV	non-invasive ventilation
NLR	neutrophil-to-lymphocyte ratio
NT-proBNP	*N*-terminal pro B-type natriuretic peptide
OCS	oral corticosteroids
RW	respiratory ward
