# Supplementary material for: Heart Failure is Highly Prevalent and Difficult to Diagnose in Severe Exacerbations of COPD Presenting to the Emergency Department
Source: J Clin Med. 2020 Aug 14;9(8):2644. doi: 10.3390/jcm9082644 (PMC7466112; doi:10.3390/jcm9082644)
Supplement: Supplementary file 1 [file jcm-09-02644-s001.pdf]

SUPPLEMENTARY DATA

Heart Failure is highly prevalent and difficult to diagnose in severe exacerbations of COPD presenting to the Emergency Department.

Mariaenrica Tinè[1*], M.D., Erica Bazzan[1*], PhD, Umberto Semenzato[1], M.D., Davide Biondini[1], M.D., PhD, Elisabetta Cocconcelli[1], M.D., Elisabetta Balestro[1], M.D., Alvise Casara[1], M.D., Simonetta Baraldo[1], PhD, Graziella Turato[1], PhD, Manuel G. Cosio[1,2**], M.D., Marina Saetta[1**], M.D.

*Drs. Tinè and Bazzan contributed equally to this article as first authors.

** Profs. Saetta and Cosio contributed equally to this article as senior authors.

Corresponding author:

Marina Saetta,

Department of Cardio-Thoracic-Vascular Sciences and Public Health, University of Padova, Via Giustiniani 3, 35128 Padova, Italy.

Email: marina.saetta@unipd.it

METHODS

Definitions of the three diagnostic AECOPD groups

EMERGENCY DEPARTMENT (ED) DIAGNOSIS

Patients presenting with acute severe exacerbation of respiratory symptoms to the ED, were diagnosed by ED physicians according to the patient's ED chart as:

1) respiratory failure secondary to only acute exacerbation of COPD (AECOPD).
2) respiratory failure secondary to AECOPD and concomitant Heart Failure (AECOPD+HF)
3) respiratory failure secondary to other diagnosis (AECOPD+OD) than AECOPD or AECOPD+HF (like pneumonia, pulmonary emboli, or "respiratory failure" with non specified cause by ED physicians).

The abbreviated form of the original ED diagnosis: AECOPD, AECOPD+HF and AECOPD+OD were used throughout the manuscript.

RESPIRATORY WARD (RW) DIAGNOSIS

A detailed diagnostic workout was performed in the RW in order to obtain the correct diagnosis in each patient.

The diagnostic denomination of AECOPD, AECOPD+HF and AECOPD+OD used in ED was maintained for the correct discharge diagnosis from the RW.

The diagnostic tests and how often were they requested by ED and RW physicians are summarized in Table S1.

Statistical analysis

Patients characteristics were described using mean±SD and counts and percentages for categorical variables. Comparisons among groups were evaluated with Kruskal-Wallis and Mann-Whitney U tests. Distributions of categorical variables were compared with the $\chi 2$-test or Fisher exact-test when the sample size was small ($n < 5$). Analyses of overall survival were performed by Kaplan-Meier survival curves. Cox proportional risk regression model was used to evaluate independent prognostic factors. Variables included in the Cox regression analysis had to be significant in the respective univariate analyses. Hazard ratios

(HRs) and 95% confidence intervals (CIs) were estimated for all the variables entered in the final model.

All analyses were performed using SPSS (version 25.0.0.1 for Windows). Statistical significance was assumed for a p value <0.05.

RESULTS

Baseline medications

The use of respiratory medications was similar in the three diagnostic groups with 86/119 (72%) using inhaled corticosteroids and bronchodilators, 19/119 (16%) using single or double bronchodilators and only 14/119 (12%) with no regular treatment.

The use of oral corticosteroids– 31/119 (26%) - and antibiotics – 43/119 (36%) – in the 2 weeks previous to hospital admission was similar in the three diagnostic categories. Among cardiovascular medications, each patient in the AECOPD+HF group used at least one: ACE inhibitors, anti-aggregants, anticoagulants, beta-blockers. Anticoagulants were more often used in patients with AECOPD+HF than AECOPD (p<0.0001) and AECOPD+OD (p=0.047) (Table S2).

Other diagnosis at the ED and RW

The group AECOPD+OD included patients with acute exacerbations of respiratory symptoms thought to be due to diagnosis other than AECOPD or AECOPD+HF. At the ED, the AECOPD+OD diagnosis included 43 patients of which: 18 were diagnosed of "respiratory failure", 9 pneumonia, 7 lung infection, 4 asthma exacerbation in patients with chronic bronchitis, 2 haemoptysis, 1 pleural effusion, 1 pulmonary embolism, 1 opioid toxicity. In the 18 patients diagnosed as respiratory failure the label "respiratory failure" was an unspecific diagnosis conferred in the absence of a clear identifiable cause for acute worsening of respiratory symptoms. In the 4 patients diagnosed as "asthma exacerbation in patients with chronic bronchitis", the presence of a history of asthma was carefully excluded at the RW evaluation.

At the RW, the AECOPD+OD discharge diagnosis was modified from the ED diagnosis and included 23 patients of which: 15 were diagnosed of pneumonia, 3 pulmonary embolism, 1

pulmonary aspergillosis, 1 opioid toxicity and 3 respiratory symptoms deterioration due to complications of advanced neoplasia.

Other diagnostic tests

A CT scan, available in 117/119 patients, showed emphysema in 53/117 (45%) and bronchiectasis in 25/117 (21%) with no difference found among the three diagnostic group. Cardiac Doppler was performed when deem indicated in 76/119 patients and heart failure was diagnosed in 48/76 according to the European Society of Cardiology guidelines [1].

Comorbidities

The evaluation of comorbidities showed that 31/119 (26%) suffered from chronic ischemic heart disease, 50/119 (42%) from chronic heart failure, 33/119 (28%) from atrial fibrillation and 81/119 (68%) from arterial hypertension. Diabetes was found in 39/119 (33%), osteoporosis in a 26/119 (22%) depressive disorder in 23/119 (19%), 20/119 (17%) had a known lung cancer and 38/119 (32%) suffered from gastro-esophageal reflux. The frequencies of comorbidities in each diagnostic group are shown in Table S3.

SUPPLEMENTARY TABLES

Table S1. Diagnostic tests available at Emergency Department (ED) and Respiratory Ward (RW) and frequency of use in the whole population of 119 patients.

	**ED** **n=119**	**RW** **n=119**
**Blood count**	100 %	100 %
**C reactive protein**	37 %	100 %
**NT-proBNP**	37 %	100 %
**Blood gas analysis**	100 %	100 %
**Chest X-rays**	100 %	100 %
**Pulmonary function test**		100 %
**Chest CT scan**		98 %
**Microbiological assays**		46 %§
**Echocardiography**		64 %

§ Bronchoscopy, serological assay and/or sputum culture.
ED=emergency department; RW=respiratory ward; NT-proBNP=N-terminal pro B-type natriuretic peptide; CT=computed tomography.

Table S2. Medications at baseline in whole population and in the three RW groups.

	Whole population	AECOPD (n=48)	AECOPD+HF (n=48)	AECOPD+OD (n=23)	p
**Respiratory medications**					
ICS/LABA n(%)	**86 (72)**	34 (71)	37 (77)	15 (66)	n.s.
LAMA or LABA/LAMA n(%)	**19 (16)**	9 (19)	6 (13)	4 (17)	n.s.
**Cardiovascular medications**					
ACE inhibitors n(%)	**42 (40)**	16 (36)	20 (47)	6 (35)	n.s.
Antiaggregants n(%)	**42 (40)**	16 (36)	20 (47)	6 (35)	n.s.
Anticoagulants n(%)	**37 (36)**	7 (16)*	25 (60)	5 (29)*	**0.0001**
Beta-blockers n(%)	**17 (16)**	4 (9)	10 (24)	3 (17)	n.s.

Data are expressed as number (%).

p values refer to chi-square test. * significantly different than AECOPD+HF ($p<0.05$). n.s.: non-significant

AECOPD=acute exacerbation of COPD; AECOPD+HF= acute exacerbation of COPD and heart failure; AECOPD+OD=diagnosis other than AECOPD or AECOPD+HF.

Table S3. Comorbidities in whole population and the three RW groups.

Comorbidity	Whole Population	AECOPD (n=48)	AECOPD+HF (n=48)	AECOPD+OD (n=23)	p
**Chronic ischemic heart disease, n (%)**	31 (26)	11 (23)	13 (27)	7 (30)	n.s.
**Chronic heart failure, n (%)**	50 (42)	10 (21)*	30 (63)	10 (43)	**0.0002**
**Atrial fibrillation, n (%)**	33 (28)	3 (6)*	23 (48)	7 (30)	**<0.0001**
**Arterial hypertension, n (%)**	81 (68)	25 (52)*§	38 (79)	18 (78)	**0.009**
**Diabetes mellitus, n (%)**	39 (33)	9 (39)*	20 (48)	10 (43)	**0.03**
**Osteoporosis, n (%)**	26 (22)	11 (23)	12 (25)	3 (13)	n.s.
**Depressive disorder, n (%)**	23 (19)	12 (25)	5 (10)	6 (26)	n.s.
**Lung cancer, n (%)**	20 (17)	8 (17)	8 (17)	4 (17)	n.s.
**Gastroesophageal reflux disease, n (%)**	38 (32)	18 (38)	14 (29)	6 (26)	n.s.

Data are expressed as number(%). p values refer to chi-square test. * significantly different than AECOPD+HF ($p<0.05$). § significantly different than AECOPD+OD ($p<0.05$). n.s.: non-significant. AECOPD=acute exacerbation of COPD; AECOPD+HF= acute exacerbation of COPD and heart failure; AECOPD+OD=diagnosis other than AECOPD or AECOPD+HF.

Table S4. Laboratory tests at ED visit according to the three RW diagnostic groups.

	**Whole Population**	**AECOPD** (n=48)	**AECOPD+HF** (n=48)	**AECOPD+OD** (n=23)	**p**
**White blood cell count, cells/μl**	11449 ± 4581	11791 ± 4227	11065 ± 4226	12423 ± 5456	n.s.
**Neutrophils, cells/μl**	8787 ± 5215	8549 ± 3898	10380 ± 4457	10801 ± 5879	n.s.
**CRP, mg/L**	82.3 ± 99.4	28.6 ± 58.5*§	93 ± 98.3	132 ± 116.3	**0.01**
**NT-proBNP, ng/L**	1596 ± 2558	144 ± 113*	2567 ± 2826	1747 ± 2987	**0.01**
**Arterial Blood Gas**					
**pH**	7.4 ± 0.1	7.39 ± 0.1	7.4 ± 0.1	7.42 ± 0.1	n.s.
**pO_2, mmHg**	64.3 ± 27.3	66.9 ± 23.5	60.7 ± 20.4	66.3 ± 43.6	n.s.
**pCO_2, mmHg**	52.1 ± 18.6	59.3 ± 18.3	52.4 ± 15.7	49.6 ± 24.8	n.s.
**$HCO3^-$, mMol/L**	30.1 ± 6.2	30.3 ± 6.5	30.8 ± 6.6	27.9 ± 4.3	n.s.

Data are expressed as mean±SD.

p values refer to Kruskal Wallis test.

*Significantly different than AECOPD+HF. § Significantly different than AECOPD+OD. n.s.: non-significant

AECOPD=acute exacerbation of COPD; AECOPD+HF= acute exacerbation of COPD and heart failure; AECOPD+OD=diagnosis other than AECOPD or AECOPD+HF; CRP=C-reactive protein; BNP=N-terminal pro B-type natriuretic peptide; pO2=partial pressure of oxygen; pCO2=partial pressure of carbon dioxide; HCO3=bicarbonate.

Table S5. Laboratory and clinical data at the RW.

	Whole Population	AECOPD (n=48)	AECOPD+HF (n=48)	AECOPD+OD (n=23)	p
**White blood cell count, cells/μl**	9777 ± 3806	10291 ± 3241	10291 ± 3809	9759 ± 3413	n.s.
**Neutrophils, cells/μl**	7624 ± 3453	7262 ± 3067	7895 ± 3598	6908 ± 2999	n.s.
**Lymphocytes, cells/μl**	1590 ± 874	1715 ± 894	1432 ± 767*	1880 ± 819	**0.05**
**Monocytes, cells/μl**	849 ± 323	819 ± 263	876 ± 331	849 ± 414	n.s.
**Eosinophils, cells/μl**	92 ± 138	110 ± 171	80 ± 110	94 ± 112	n.s.
**Basophils, cells/μl**	23 ± 47	49 ± 155	13 ± 17	32 ± 47	n.s.
**NLR**	5.2 ± 7.1	6.5 ± 7.5	7.7 ± 7.9	4.3 ± 2.4	n.s.
**Length of stay, days**	9 ± 6	8 ± 5	9 ± 5	11 ± 7	n.s.
**NIV, n (%)**	50 (42)	15 (31)	28 (58)§	7 (30)	**<0.0001**
**Infective exacerbations, n(%)**	38/55 (69)	17/22 (77)	9/16 (56)	12/17 (70)	n.s.
**Bacteria, n (%)**	14/38 (37)	8/17 (47)	3/9 (33)	4/12 (33)	n.s.
**Virus, n (%)**	8/38 (21)	5/17 (29)	2/9 (23)	1/12 (8)	n.s.
**Fungi, n (%)**	10/38 (26)	3/17 (18)	3/9 (33)	4/12 (33)	n.s.
**Mix°, n (%)**	6/38 (16)	1/17 (6)	1/9 (11)	3/12 (24)	n.s.

Data are expressed as number(%) or mean±SD.

p values refer to Kruskal Wallis test and chi-square test. * significantly different than AECOPD+OE ($p<0.05$). § significantly different than AECOPD and AECOPD+OD ($p<0.05$).

AECOPD=acute exacerbation of COPD; AECOPD+HF= acute exacerbation of COPD and heart failure; AECOPD+OD= diagnosis other than AECOPD or AECOPD+HF; NLR= neutrophil-to lymphocytes ratio; NIV=non-invasive ventilation.

° The mixed positive microbiologic assays in the 3 groups:

AECOPD: Acinetobacter Baumanni + mycobacterium intracellulare + Respiratory Syncytial Virus (RSV). AECOPD+HF: Candida Albicans + RSV. AECOPD+OD: Staphylococcus Aureus + Cytomegalovirus (CMV); Chlamydia + C. Albicans; Stenotrophomonas Maltophila + CMV.

Table S6. Risk factors for death after discharge from our RW, non-adjusted and adjusted HR.

Variable	Univariate analysis		Multivariate analysis	
	HR (95% CI)	p	(95% CI)	p
**Age (*yrs*)**	1.07 (1.04-1.11)	**<0.001**	1.06 (1.02-1.09)	**0.002**
**Sex (female)**	1.4 (0.84-2.33)	0.19	-	-
**FEV_1 (% pred)**	1 (0.98-1.02)	0.74	-	-
**GOLD stage 3-4**	1.78 (1.04-3.07)	**0.036**	-	-
**pCO_2 (*mmHg*) §**	1 (0.99-1.02)	0.63	-	-
**C-reactive protein (*mg/L*) §**	0.99 (0.99-1)	0.44	-	-
**NT-proBNP§ (*ng/L*)**	1 (1-1)	0.39	-	-
**Heart failure**	2.58 (1.54-4.31)	**0.0003**	2.19 (1.26-3.79)	**0.005**
**Early readmission**	2.08 (1.24 – 3.49)	**0.006**	2.05 (1.11-3.79)	**0.02**
**No COPD care program**	2.65 (1.5-4.71)	**0.001**	2.56 (1.31-4.99)	**0.006**
**Leukocytes* - n $x10^9$/L**	0.95 (0.88-1.02)	0.17	-	-
**Neutrophils* - n $x10^9$/L**	0.99 (0.91-1.07)	0.79	-	-
**Lymphocytes* - n $x10^9$/L**	0.51 (0.36-0.74)	**0.0003**	0.57 (0.37-0.87)	**0.01**
**Eosinophils* - n $x10^9$/L**	0.12 (0.01-1.42)	0.09	-	-
**Neutrophil-to-lymphocyte ratio***	1.03 (1-1.06)	**0.02**	-	-

Values are expressed as HR (95%CI). Univariate and multivariate Cox proportional hazard regression tests were used to determine the relationship of clinical, functional and serological characteristics with survival.

§ Lab tests refer to ED visit median and positive values.

*Lab tests refer to RW discharge median values.

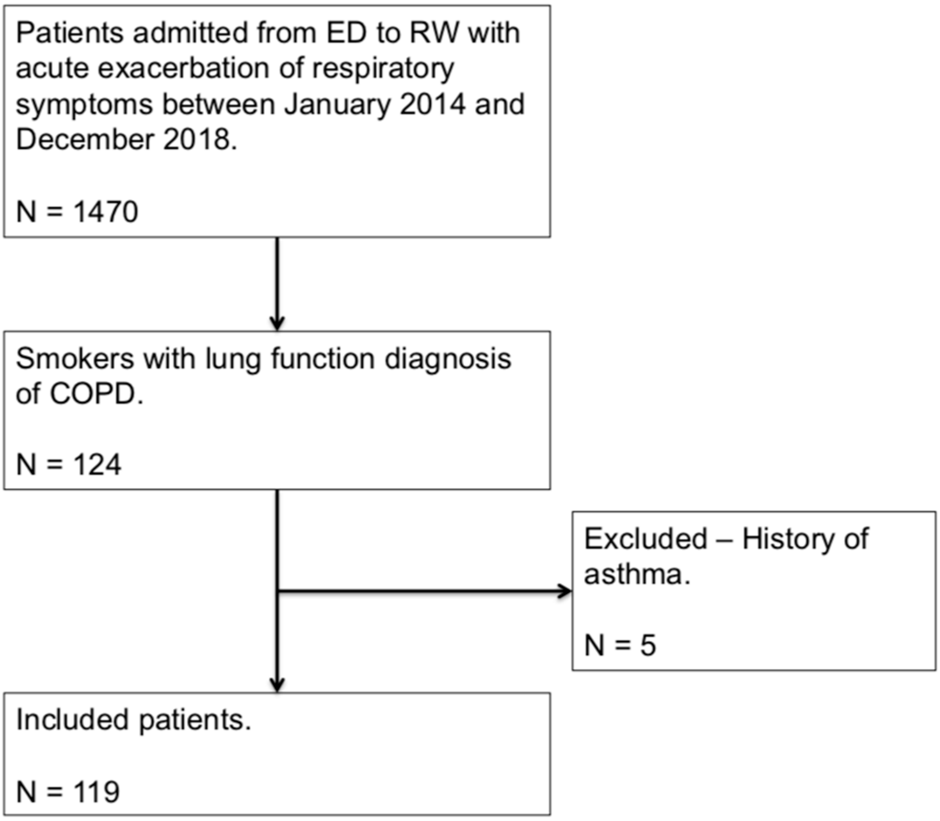

Figure S1: Patient flow chart

SUPPLEMENTARY REFERENCES

[1]. Ponikowski P, Voors AA, Anker SD, Bueno H, Cleland JG, Coats AJ, Falk V, González-Juanatey JR, Harjola VP, Jankowska EA, Jessup M, Linde C, Nihoyannopoulos P, Parissis JT, Pieske B, Riley JP, Rosano GM, Ruilope LM, Ruschitzka F, Rutten FH, van der Meer P; Authors/Task Force Members; Document Reviewers. 2016 ESC Guidelines for the diagnosis and treatment of acute and chronic heart failure: The Task Force for the diagnosis and treatment of acute and chronic heart failure of the European Society of Cardiology (ESC). Developed with the special contribution of the Heart Failure Association (HFA) of the ESC. Eur J Heart Fail. 2016 Aug;18(8):891
